# Supplementary material for: A fault-intrusion-tolerant system and deadline-aware algorithm for scheduling scientific workflow in the cloud
Source: PeerJ Comput Sci. 2021 Nov 2;7:e747. doi: 10.7717/peerj-cs.747 (PMC8576554; doi:10.7717/peerj-cs.747)
Supplement: Supplemental Information 6 [file peerj-cs-07-747-s006.zip › Myproject1.1/docs/org/cloudbus/cloudsim/power/PowerVmAllocationPolicyMigrationAbstract.html]

PowerVmAllocationPolicyMigrationAbstract


JavaScript is disabled on your browser.


- Overview
- Package
- Class
- Use
- Tree
- Deprecated
- Index
- Help

- Prev Class
- Next Class

- Frames
- No Frames

- All Classes

- Summary:
- Nested |
- Field |
- Constr |
- Method

- Detail:
- Field |
- Constr |
- Method


org.cloudbus.cloudsim.power

## Class PowerVmAllocationPolicyMigrationAbstract

- java.lang.Object
- - org.cloudbus.cloudsim.VmAllocationPolicy
  - - org.cloudbus.cloudsim.power.PowerVmAllocationPolicyAbstract
    - - org.cloudbus.cloudsim.power.PowerVmAllocationPolicyMigrationAbstract

- Direct Known Subclasses:
  :   PowerVmAllocationPolicyMigrationInterQuartileRange, PowerVmAllocationPolicyMigrationLocalRegression, PowerVmAllocationPolicyMigrationMedianAbsoluteDeviation, PowerVmAllocationPolicyMigrationStaticThreshold

  ---

    

  ```
  public abstract class PowerVmAllocationPolicyMigrationAbstract
  extends PowerVmAllocationPolicyAbstract
  ```

  The class of an abstract power-aware VM allocation policy that dynamically optimizes the VM
  allocation using migration.
  If you are using any algorithms, policies or workload included in the power package, please cite
  the following paper:
  Anton Beloglazov, and Rajkumar Buyya, "Optimal Online Deterministic Algorithms and Adaptive
  Heuristics for Energy and Performance Efficient Dynamic Consolidation of Virtual Machines in
  Cloud Data Centers", Concurrency and Computation: Practice and Experience (CCPE), Volume 24,
  Issue 13, Pages: 1397-1420, John Wiley & Sons, Ltd, New York, USA, 2012

  Since:
  :   CloudSim Toolkit 3.0

- - ### Constructor Summary

    Constructors

    Constructor and Description

    `PowerVmAllocationPolicyMigrationAbstract(java.util.List<? extends Host> hostList,
    PowerVmSelectionPolicy vmSelectionPolicy)`

    Instantiates a new power vm allocation policy migration abstract.
  - ### Method Summary

    Methods

    | Modifier and Type | Method and Description |
    | --- | --- |
    | `protected void` | `addHistoryEntry(HostDynamicWorkload host, double metric)` Adds the history value. |
    | `protected boolean` | `areAllVmsMigratingOutOrAnyVmMigratingIn(PowerHost host)` Checks whether all vms are in migration. |
    | `protected java.util.List<PowerHost>` | `extractHostListFromMigrationMap(java.util.List<java.util.Map<java.lang.String,java.lang.Object>> migrationMap)` Extract host list from migration map. |
    | `PowerHost` | `findHostForVm(Vm vm)` Find host for vm. |
    | `PowerHost` | `findHostForVm(Vm vm, java.util.Set<? extends Host> excludedHosts)` Find host for vm. |
    | `java.util.List<java.lang.Double>` | `getExecutionTimeHistoryHostSelection()` Gets the execution time history host selection. |
    | `java.util.List<java.lang.Double>` | `getExecutionTimeHistoryTotal()` Gets the execution time history total. |
    | `java.util.List<java.lang.Double>` | `getExecutionTimeHistoryVmReallocation()` Gets the execution time history vm reallocation. |
    | `java.util.List<java.lang.Double>` | `getExecutionTimeHistoryVmSelection()` Gets the execution time history vm selection. |
    | `protected double` | `getMaxUtilizationAfterAllocation(PowerHost host, Vm vm)` Gets the power after allocation. |
    | `java.util.Map<java.lang.Integer,java.util.List<java.lang.Double>>` | `getMetricHistory()` Gets the metric history. |
    | `protected java.util.List<java.util.Map<java.lang.String,java.lang.Object>>` | `getMigrationMapFromUnderUtilizedHosts(java.util.List<PowerHostUtilizationHistory> overUtilizedHosts)` Gets the migration map from under utilized hosts. |
    | `protected java.util.List<java.util.Map<java.lang.String,java.lang.Object>>` | `getNewVmPlacement(java.util.List<? extends Vm> vmsToMigrate, java.util.Set<? extends Host> excludedHosts)` Gets the new vm placement. |
    | `protected java.util.List<java.util.Map<java.lang.String,java.lang.Object>>` | `getNewVmPlacementFromUnderUtilizedHost(java.util.List<? extends Vm> vmsToMigrate, java.util.Set<? extends Host> excludedHosts)` Gets the new vm placement from under utilized host. |
    | `protected java.util.List<PowerHostUtilizationHistory>` | `getOverUtilizedHosts()` Gets the over utilized hosts. |
    | `protected double` | `getPowerAfterAllocation(PowerHost host, Vm vm)` Gets the power after allocation. |
    | `protected java.util.List<java.util.Map<java.lang.String,java.lang.Object>>` | `getSavedAllocation()` Gets the saved allocation. |
    | `protected java.util.List<PowerHost>` | `getSwitchedOffHosts()` Gets the switched off host. |
    | `java.util.Map<java.lang.Integer,java.util.List<java.lang.Double>>` | `getTimeHistory()` Gets the time history. |
    | `protected PowerHost` | `getUnderUtilizedHost(java.util.Set<? extends Host> excludedHosts)` Gets the under utilized host. |
    | `java.util.Map<java.lang.Integer,java.util.List<java.lang.Double>>` | `getUtilizationHistory()` Gets the utilization history. |
    | `protected double` | `getUtilizationOfCpuMips(PowerHost host)` Gets the utilization of the CPU in MIPS for the current potentially allocated VMs. |
    | `protected PowerVmSelectionPolicy` | `getVmSelectionPolicy()` Gets the vm selection policy. |
    | `protected java.util.List<? extends Vm>` | `getVmsToMigrateFromHosts(java.util.List<PowerHostUtilizationHistory> overUtilizedHosts)` Gets the vms to migrate from hosts. |
    | `protected java.util.List<? extends Vm>` | `getVmsToMigrateFromUnderUtilizedHost(PowerHost host)` Gets the vms to migrate from under utilized host. |
    | `protected abstract boolean` | `isHostOverUtilized(PowerHost host)` Checks if is host over utilized. |
    | `protected boolean` | `isHostOverUtilizedAfterAllocation(PowerHost host, Vm vm)` Checks if is host over utilized after allocation. |
    | `java.util.List<java.util.Map<java.lang.String,java.lang.Object>>` | `optimizeAllocation(java.util.List<? extends Vm> vmList)` Optimize allocation of the VMs according to current utilization. |
    | `protected void` | `printOverUtilizedHosts(java.util.List<PowerHostUtilizationHistory> overUtilizedHosts)` Prints the over utilized hosts. |
    | `protected void` | `restoreAllocation()` Restore allocation. |
    | `protected void` | `saveAllocation()` Save allocation. |
    | `protected void` | `setVmSelectionPolicy(PowerVmSelectionPolicy vmSelectionPolicy)` Sets the vm selection policy. |

    - ### Methods inherited from class org.cloudbus.cloudsim.power.PowerVmAllocationPolicyAbstract

      `allocateHostForVm, allocateHostForVm, deallocateHostForVm, getHost, getHost, getVmTable`
    - ### Methods inherited from class org.cloudbus.cloudsim.VmAllocationPolicy

      `getHostList, setHostList`
    - ### Methods inherited from class java.lang.Object

      `clone, equals, finalize, getClass, hashCode, notify, notifyAll, toString, wait, wait, wait`

- - ### Constructor Detail


    - #### PowerVmAllocationPolicyMigrationAbstract

      ```
      public PowerVmAllocationPolicyMigrationAbstract(java.util.List<? extends Host> hostList,
                                              PowerVmSelectionPolicy vmSelectionPolicy)
      ```

      Instantiates a new power vm allocation policy migration abstract.

      Parameters:
      :   `hostList` - the host list
      :   `vmSelectionPolicy` - the vm selection policy
  - ### Method Detail


    - #### optimizeAllocation

      ```
      public java.util.List<java.util.Map<java.lang.String,java.lang.Object>> optimizeAllocation(java.util.List<? extends Vm> vmList)
      ```

      Optimize allocation of the VMs according to current utilization.

      **Specified by:**
      :   `optimizeAllocation` in class `VmAllocationPolicy`

      Parameters:
      :   `vmList` - the vm list

      Returns:
      :   the array list< hash map< string, object>>


    - #### getMigrationMapFromUnderUtilizedHosts

      ```
      protected java.util.List<java.util.Map<java.lang.String,java.lang.Object>> getMigrationMapFromUnderUtilizedHosts(java.util.List<PowerHostUtilizationHistory> overUtilizedHosts)
      ```

      Gets the migration map from under utilized hosts.

      Parameters:
      :   `overUtilizedHosts` - the over utilized hosts

      Returns:
      :   the migration map from under utilized hosts


    - #### printOverUtilizedHosts

      ```
      protected void printOverUtilizedHosts(java.util.List<PowerHostUtilizationHistory> overUtilizedHosts)
      ```

      Prints the over utilized hosts.

      Parameters:
      :   `overUtilizedHosts` - the over utilized hosts


    - #### findHostForVm

      ```
      public PowerHost findHostForVm(Vm vm,
                            java.util.Set<? extends Host> excludedHosts)
      ```

      Find host for vm.

      Parameters:
      :   `vm` - the vm
      :   `excludedHosts` - the excluded hosts

      Returns:
      :   the power host


    - #### isHostOverUtilizedAfterAllocation

      ```
      protected boolean isHostOverUtilizedAfterAllocation(PowerHost host,
                                              Vm vm)
      ```

      Checks if is host over utilized after allocation.

      Parameters:
      :   `host` - the host
      :   `vm` - the vm

      Returns:
      :   true, if is host over utilized after allocation


    - #### findHostForVm

      ```
      public PowerHost findHostForVm(Vm vm)
      ```

      Find host for vm.

      **Overrides:**
      :   `findHostForVm` in class `PowerVmAllocationPolicyAbstract`

      Parameters:
      :   `vm` - the vm

      Returns:
      :   the power host


    - #### extractHostListFromMigrationMap

      ```
      protected java.util.List<PowerHost> extractHostListFromMigrationMap(java.util.List<java.util.Map<java.lang.String,java.lang.Object>> migrationMap)
      ```

      Extract host list from migration map.

      Parameters:
      :   `migrationMap` - the migration map

      Returns:
      :   the list


    - #### getNewVmPlacement

      ```
      protected java.util.List<java.util.Map<java.lang.String,java.lang.Object>> getNewVmPlacement(java.util.List<? extends Vm> vmsToMigrate,
                                                                                       java.util.Set<? extends Host> excludedHosts)
      ```

      Gets the new vm placement.

      Parameters:
      :   `vmsToMigrate` - the vms to migrate
      :   `excludedHosts` - the excluded hosts

      Returns:
      :   the new vm placement


    - #### getNewVmPlacementFromUnderUtilizedHost

      ```
      protected java.util.List<java.util.Map<java.lang.String,java.lang.Object>> getNewVmPlacementFromUnderUtilizedHost(java.util.List<? extends Vm> vmsToMigrate,
                                                                                                            java.util.Set<? extends Host> excludedHosts)
      ```

      Gets the new vm placement from under utilized host.

      Parameters:
      :   `vmsToMigrate` - the vms to migrate
      :   `excludedHosts` - the excluded hosts

      Returns:
      :   the new vm placement from under utilized host


    - #### getVmsToMigrateFromHosts

      ```
      protected java.util.List<? extends Vm> getVmsToMigrateFromHosts(java.util.List<PowerHostUtilizationHistory> overUtilizedHosts)
      ```

      Gets the vms to migrate from hosts.

      Parameters:
      :   `overUtilizedHosts` - the over utilized hosts

      Returns:
      :   the vms to migrate from hosts


    - #### getVmsToMigrateFromUnderUtilizedHost

      ```
      protected java.util.List<? extends Vm> getVmsToMigrateFromUnderUtilizedHost(PowerHost host)
      ```

      Gets the vms to migrate from under utilized host.

      Parameters:
      :   `host` - the host

      Returns:
      :   the vms to migrate from under utilized host


    - #### getOverUtilizedHosts

      ```
      protected java.util.List<PowerHostUtilizationHistory> getOverUtilizedHosts()
      ```

      Gets the over utilized hosts.

      Returns:
      :   the over utilized hosts


    - #### getSwitchedOffHosts

      ```
      protected java.util.List<PowerHost> getSwitchedOffHosts()
      ```

      Gets the switched off host.

      Returns:
      :   the switched off host


    - #### getUnderUtilizedHost

      ```
      protected PowerHost getUnderUtilizedHost(java.util.Set<? extends Host> excludedHosts)
      ```

      Gets the under utilized host.

      Parameters:
      :   `excludedHosts` - the excluded hosts

      Returns:
      :   the under utilized host


    - #### areAllVmsMigratingOutOrAnyVmMigratingIn

      ```
      protected boolean areAllVmsMigratingOutOrAnyVmMigratingIn(PowerHost host)
      ```

      Checks whether all vms are in migration.

      Parameters:
      :   `host` - the host

      Returns:
      :   true, if successful


    - #### isHostOverUtilized

      ```
      protected abstract boolean isHostOverUtilized(PowerHost host)
      ```

      Checks if is host over utilized.

      Parameters:
      :   `host` - the host

      Returns:
      :   true, if is host over utilized


    - #### addHistoryEntry

      ```
      protected void addHistoryEntry(HostDynamicWorkload host,
                         double metric)
      ```

      Adds the history value.

      Parameters:
      :   `host` - the host
      :   `metric` - the metric


    - #### saveAllocation

      ```
      protected void saveAllocation()
      ```

      Save allocation.


    - #### restoreAllocation

      ```
      protected void restoreAllocation()
      ```

      Restore allocation.


    - #### getPowerAfterAllocation

      ```
      protected double getPowerAfterAllocation(PowerHost host,
                                   Vm vm)
      ```

      Gets the power after allocation.

      Parameters:
      :   `host` - the host
      :   `vm` - the vm

      Returns:
      :   the power after allocation


    - #### getMaxUtilizationAfterAllocation

      ```
      protected double getMaxUtilizationAfterAllocation(PowerHost host,
                                            Vm vm)
      ```

      Gets the power after allocation. We assume that load is balanced between PEs. The only
      restriction is: VM's max MIPS < PE's MIPS

      Parameters:
      :   `host` - the host
      :   `vm` - the vm

      Returns:
      :   the power after allocation


    - #### getUtilizationOfCpuMips

      ```
      protected double getUtilizationOfCpuMips(PowerHost host)
      ```

      Gets the utilization of the CPU in MIPS for the current potentially allocated VMs.

      Parameters:
      :   `host` - the host

      Returns:
      :   the utilization of the CPU in MIPS


    - #### getSavedAllocation

      ```
      protected java.util.List<java.util.Map<java.lang.String,java.lang.Object>> getSavedAllocation()
      ```

      Gets the saved allocation.

      Returns:
      :   the saved allocation


    - #### setVmSelectionPolicy

      ```
      protected void setVmSelectionPolicy(PowerVmSelectionPolicy vmSelectionPolicy)
      ```

      Sets the vm selection policy.

      Parameters:
      :   `vmSelectionPolicy` - the new vm selection policy


    - #### getVmSelectionPolicy

      ```
      protected PowerVmSelectionPolicy getVmSelectionPolicy()
      ```

      Gets the vm selection policy.

      Returns:
      :   the vm selection policy


    - #### getUtilizationHistory

      ```
      public java.util.Map<java.lang.Integer,java.util.List<java.lang.Double>> getUtilizationHistory()
      ```

      Gets the utilization history.

      Returns:
      :   the utilization history


    - #### getMetricHistory

      ```
      public java.util.Map<java.lang.Integer,java.util.List<java.lang.Double>> getMetricHistory()
      ```

      Gets the metric history.

      Returns:
      :   the metric history


    - #### getTimeHistory

      ```
      public java.util.Map<java.lang.Integer,java.util.List<java.lang.Double>> getTimeHistory()
      ```

      Gets the time history.

      Returns:
      :   the time history


    - #### getExecutionTimeHistoryVmSelection

      ```
      public java.util.List<java.lang.Double> getExecutionTimeHistoryVmSelection()
      ```

      Gets the execution time history vm selection.

      Returns:
      :   the execution time history vm selection


    - #### getExecutionTimeHistoryHostSelection

      ```
      public java.util.List<java.lang.Double> getExecutionTimeHistoryHostSelection()
      ```

      Gets the execution time history host selection.

      Returns:
      :   the execution time history host selection


    - #### getExecutionTimeHistoryVmReallocation

      ```
      public java.util.List<java.lang.Double> getExecutionTimeHistoryVmReallocation()
      ```

      Gets the execution time history vm reallocation.

      Returns:
      :   the execution time history vm reallocation


    - #### getExecutionTimeHistoryTotal

      ```
      public java.util.List<java.lang.Double> getExecutionTimeHistoryTotal()
      ```

      Gets the execution time history total.

      Returns:
      :   the execution time history total


- Overview
- Package
- Class
- Use
- Tree
- Deprecated
- Index
- Help

- Prev Class
- Next Class

- Frames
- No Frames

- All Classes

- Summary:
- Nested |
- Field |
- Constr |
- Method

- Detail:
- Field |
- Constr |
- Method
